# Increased Inequalities in Health Resource and Access to Health Care in Rural China

**DOI:** 10.3390/ijerph16010049

**Published:** 2018-12-25

**Authors:** Suhang Song, Beibei Yuan, Luyu Zhang, Gang Cheng, Weiming Zhu, Zhiyuan Hou, Li He, Xiaochen Ma, Qingyue Meng

**Affiliations:** 1 School of Public Health, Peking University, Beijing 100191, China; suhangsong@126.com (S.S.); lulilu@sina.com (L.Z.); 2 China Center for Health Development Studies, Peking University, Beijing 100191, China; beibeiyuan@bjmu.edu.cn (B.Y.); maxdeapro@gmail.com (G.C.); epizhu@gmail.com (W.Z.); xma@hsc.pku.edu.cn (X.M.); 3 School of Public Health, Fudan University, Shanghai 200032, China; zyhou@fudan.edu.cn; 4 College of Physical Education and Sports, Beijing Normal University, Beijing 100875, China; aprilhelly@bjmu.edu.cn

**Keywords:** health care reform, inequality, health resource, access, China

## Abstract

Both health resources and access to these resources increased after China’s health care reform launched in 2009. However, it is not clear if the inequalities were reduced within rural China, which was one of the main targets in the reform. This study aims to examine the changes in inequalities in health resources and access following the reform. Data came from the routine report of rural counties in every other year from 2008 to 2014. Health professionals and hospital beds per 1000 population were used for measuring health resources, and the hospitalization rate was used for access. Descriptive analysis and the fixed effect model were used in this study. Health resources and access increased by about 50% between 2008 and 2014 in rural China. The counties in richer quintiles got more health resources and hospitalizations. As for health professionals, the absolute differences between the richer and the poorest quintile were significantly enlarging in 2014 when compared to 2008. Regarding the hospitalization rate, the differences between the richest and the poorest quintile showed no significant change after 2012. In sum, absolute inequalities of health resources were increased, while that of health utilization kept constant following China’s health care reform. The reform needs to continually recruit qualified health workers and appropriately allocate health infrastructures to strengthen the capacity of the health care system in the impoverished areas.

## 1. Introduction

Reducing inequality in health and health care is one of the expected outcomes in China’s current health care reform, which was initiated in 2009 and is aiming to achieve universal health coverage by 2020 [1]. Policies and interventions for a more equitable health system were implemented in this reform, including strengthening the primary health care (PHC) system, expanding health insurance coverage through increasing subsidies to the poor areas (western and central regions) by the central government, and providing government subsidies for public health services focusing on the poor areas [2]. A special education program was implemented for improving the availability of the human resources for rural areas, especially the poor areas, in which tuition fees are exempt for the medical students who work in the rural areas after graduation [1]. The central government has increased financial transfer payments to support the poor areas in developing their local health system. Tax rebates and transfer payments in the general public budget from the central government to the local governments was 5509.75 billion Chinese yuan (847.65 billion US dollars) in 2015, and the low-income provinces (western and central regions) accounted for 76.9% (4236.79 billion Chinese yuan, 651.81 billion US dollars) [3]. During the first six years of the reform—between 2009 and 2015—the central government doubled the health budgets. Most of the budgets were used for the low-income provinces. However, improving the inequality in health care is challenging during the economic transition and the epidemiologic transition, especially since China’s Gini index has always been higher than 0.4 despite decreasing from 0.491 in 2008 to 0.469 in 2014, which shows that China has been going through an extremely inequitable economic society. Gini index in China was higher than many other Asian countries and low- and middle- income countries (LMICs), such as Japan (0.32 in 2008), South Korea (0.31 in 2007), India (0.33 in 2012), and Russia (0.41 in 2012). 

Health resource is one of the indicators for measuring the capacity of the health care system, and health resource can determine the access to health care from the supply side. A positive relationship between health resource and access to health care can be found in previous research [4,5,6,7]. To reduce the gap in health resource between poor and non-poor areas is crucial for improving the inequality in access to health care. Equitable health resource and access can help people get out of poor health status and poverty [8,9,10,11]. Overall, the numbers of health professionals per 1000 population were 3.81 in 2008 and increased to 5.56 in 2014. Meanwhile, the number of hospital beds per 1000 population increased from 3.05 in 2008 to 4.85 in 2014 [12,13]. Consequently, health care utilization also rapidly increased. Outpatient visits increased by 55.1% and inpatient care increased by 78.0% between 2008 and 2014 [12,13]. With these positive changes, it is still unclear whether the inequalities in health resource and access to health care are improved or not. 

Although some previous studies analyzed the distribution of health workforce and hospital beds in western and rural areas [14,15,16,17,18,19], most of those studies were cross-sectional studies and only focused on some areas instead of the national sample. These results cannot represent the whole of China. This research did not examine the changes in health resource and access to health care as well as their inequalities following China’s health care reform. Also, analysis about China’s health inequality mainly focused on the dimensions of urban/rural, regions (eastern, central, and western), and income quintiles, rather than the inequalities among the rural counties. However, there are about 2000 rural counties in China, which account for two out of every three county-level administrative units in total. Income gaps in these rural counties are wider than those in the urban districts. The highest province-level disposable income for rural residents was 2.31 times greater than the lowest, whereas the difference was only 1.72 times for urban residents’ disposable income [20]. This imbalanced economic development may result in an imbalanced health input and may cause differential health access and health status. Besides, according to the national strategy of poverty alleviation, taking targeted measures in vulnerable areas, like the rural areas, is of great importance. Thus, this study only focused on rural China, excluding the urban districts. Also, as the county is an important level in China’s administrative hierarchy, almost all policies of health care are tailored at county-level administrative units, and county-level inequality analysis would offer more precise policy evidence. Empirical experience in rural China may also contribute to the LMICs.

In this paper, we focused on the inequality of health resources and access to health care in rural China. We assembled a county-level longitudinal dataset, which consisted of all county-level administrative units in rural China from every other year of 2008 to 2014. Descriptive analysis and the fixed effect model were used to measure and analyze changes in the inequalities of health professionals, hospital beds, and the hospitalization rate over time. Our work could produce direct evidence for how much the inequality has changed, which would help to develop health care policies and achieve an equitable health system.

## 2. Methods 

### 2.1. Data Sources

County-level data were used in this study, which covered almost all rural counties of the 31 provinces in 2008, 2010, 2012, and 2014. County-level administrative units consist of county-level cities, counties, autonomous counties, banners, autonomous banners, forestry districts, and special districts. The urban districts were not included in this study. We have assembled a dataset by merging two sources of data. The first source included the number of health professionals, the number of hospital beds, and the number of inpatients, and was obtained from the routine annual reports of each county by the National Health Commission, which contained both the county-level administrative units and the urban districts. The second data source was the county-level GDP (Gross Domestic Product) and population data from each county’s routine statistical report, obtained from the National Bureau of Statistics and the Bureau of Statistics in each province, which contained 2009 county-level administrative units in 2008, 2010, 2012, and 2014. 

In this study, data from 1978 rural counties in 2014 were used after merging these two data sources. The numbers of counties and covered population are reported in Table 1. Because some counties have missed reporting health resources and GDP or population data, some have changed their names or zip codes, some have been combined into one county, some have been merged into other counties or districts, and some have become the urban districts. The number of counties was different in each year. Not included in this study were 102 counties (5.2%), 169 counties (8.5%), and 89 counties (4.5%) in 2008, 2010, and 2012, respectively, compared to the year of 2014. In 2014, 957.4 million people were covered, accounting for 70.0% of the total population in China. 

All rural counties were grouped into five income quintiles according to their GDP per capita. The bottom quintile (1st quintile) was the poorest counties, while the top quintile (5th quintile) was the richest counties. Three variables were used for measuring health resources and access to health care: the density of health professionals (health professionals per 1000 population), the density of hospital beds (hospital beds per 1000 population), and the hospitalization rate (inpatients per 100 population) in each year.

### 2.2. Data Analysis

We used descriptive analysis and the fixed effect model to measure the inequalities in health resource and access to health care.

After the Hausman test, the fixed effect model was chosen to measure the impact of the economic level, the time and the interaction effect of economic level, and the time on health resource and access to health care. This model compared the mean of each health indicator in different groups and also tested the time trend of the gaps between groups. The dependent variable was the value of the three variables (the density of health professionals, the density of hospital beds, and the hospitalization rate). The general formulas were [21]:

(1) $$Y_{\mathrm{ij}}=\alpha_{0}+\overset{5}{\underset{i=2}{\sum }}\beta_{i}Q_{i}+\overset{6}{\underset{j=2}{\sum }}\delta_{j}T_{j}+\overset{5}{\underset{i=2}{\sum }}\overset{6}{\underset{j=2}{\sum }}\gamma_{ij}Q_{i}*T_{j}+\mu_{i}+\varepsilon_{ij}$$

where $Q_{j}$ was a dummy variable of GDP per capita quintiles ($Q_{j}$ = 1, 2, 3, 4, and 5, if GDP per capita quintile was 1st, 2nd, 3rd, 4th, and 5th) and $T_{j}$ was a dummy variable of years ($T_{j}$ = 2008, 2010, 2012, and 2014). We set the 1st GDP per capita quintile and the year 2008 as the reference group. $\beta_{i}$ showed the gaps of different GDP per capita quintiles with the 1st quintile in the year 2008; $\delta_{i}$ showed the differences of the years 2010, 2012, and 2014 with the year 2008 in the 1st GDP per capita quintile; and $\gamma_{ij}$ showed the change of differences between the four richer quintiles and the poorest quintile in the latter years compared to 2008.

## 3. Results

In this section, we present the results of the descriptive analysis in Section 3.1, Section 3.2 and Section 3.3, and the fixed effect model in Section 3.4.

### 3.1. Description of the Inequality in Health Professionals per 1000 Population

**Figure 1:**
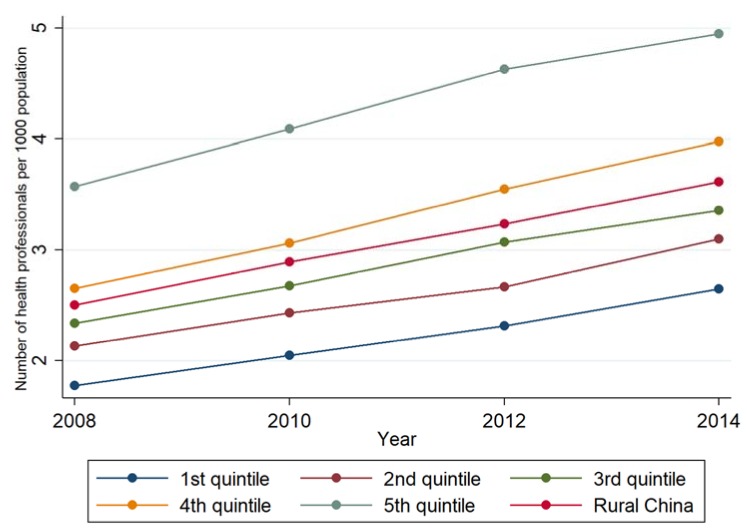
The density of health professionals by GDP per capita quintile, 2008–2014.

On average, the density of health professionals increased by nearly 50% between 2008 (one year before the health care reform) and 2014 (five years after the health care reform). The increment was larger in the poorest counties (the 1st quintile, 49.4%) than that in the richest counties (the 5th quintile, 38.7%). There was a growing inequality measured by the absolute difference (the gap between the 5th quintile and the 1st quintile was increased from 1.8 in 2008 to 2.3 in 2014). Numbers of health professionals per 1000 population in the 4th and the 5th quintiles remained higher than overall rural China during this time (Figure 1).

### 3.2. Description of the Inequality in Hospital Beds per 1000 Population

**Figure 2:**
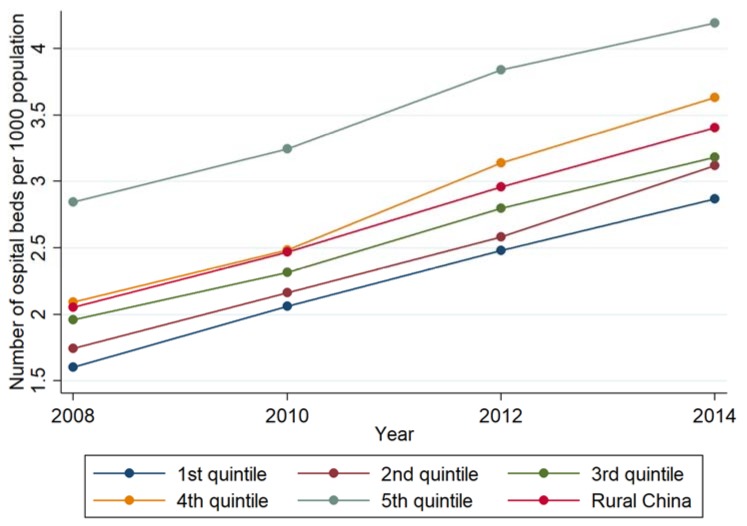
The density of hospital beds by GDP per capita quintile, 2008–2014.

On average, the density of hospital beds increased by nearly 50% to 80% between 2008 and 2014. The increment was larger in the poorest counties (the 1st quintile, 79.1%) than the richest counties (the 5th quintile, 47.3%). The inequality increased slightly and was measured by the absolute difference (from 1.2 in the year 2008 to 1.3 in the year 2014). As with the density of health professionals, the number of hospital beds per 1000 population in the 4th and the 5th quintiles remained higher than overall rural China during this time (Figure 2).

### 3.3. Description of the Inequality in the Hospitalization Rate

**Figure 3:**
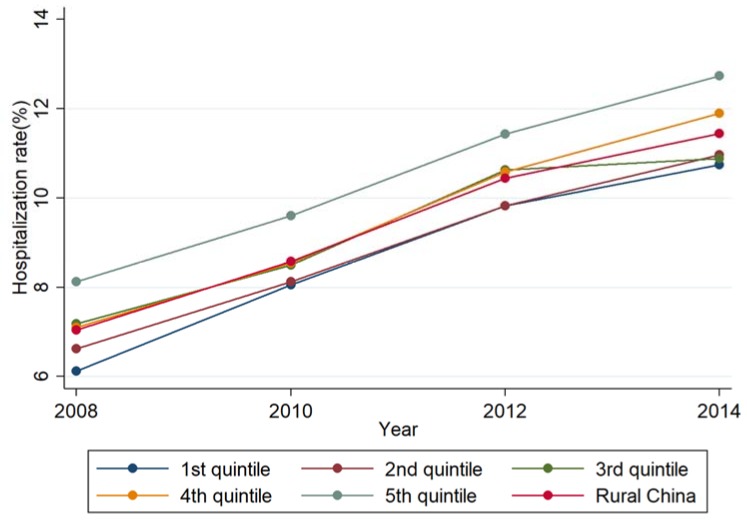
The hospitalization rate by GDP per capita quintile, 2008–2014.

On average, the hospitalization rate increased by over 50% from 2008 to 2014. The increment was larger in the poorest counties (the 1st quintile, 75.5%) than the richest counties (the 5th quintile, 56.7%). The absolute difference of the hospitalization rate fluctuated from 2.0 percentage points in 2008 to 1.5 percentage points in 2010, and then got back to 2.0 percentage points in 2014, with nearly no change between 2008 and 2014. The hospitalization rate in the 3rd and the 4th quintiles were similar to overall rural China from 2008 to 2012. The hospitalization rate in the 4th quintile was higher than overall rural China in 2014, and the hospitalization rate in the 3rd quintile was near the 1st and 2nd quintiles with a slow growth in 2014. The hospitalization rate in the 5th quintile remained higher during this time. (Figure 3).

### 3.4. Changes in the Inequality in Health Resource and Access to Health Care

In general, Model 1 showed that the number of health professionals increased by 0.308, 0.498, and 0.865 per 1000 population with statistical significance in the 1st GDP per capita quintile in 2010, 2012, and 2014 respectively when compared to 2008. Significantly, the 3rd quintile had 0.152 more health professionals per 1000 population than the 1st quintile in 2008. The absolute differences between the richer quintiles and the poorest quintile were significantly enlarging in 2014. To be more specific, the gap between the 5th quintile and the 1st quintile widened by 0.249, 0.667, and 0.754 health professionals per 1000 population in 2010, 2012, and 2014, respectively. Additionally, the differences between the 3rd and 4th quintiles and the 1st also enlarged in 2012 and 2014. Moreover, the gap between the 2nd quintile and the 1st widened in 2014 (Table 2).

Model 2 showed that hospital beds significantly increased by 0.468, 0.892, and 1.328 per 1000 population in the 1st quintile in 2010, 2012, and 2014, compared to 2008. The 2nd, 3rd, and 4th quintiles had 0.161, 0.271, and 0.175 more hospital beds per 1000 population than the 1st quintile in 2008. The absolute differences between the 5th quintile and the poorest quintile were significantly enlarging by 0.204 and 0.169 hospital beds per 1000 population in 2012 and 2014, respectively. The gap between the 4th quintile and the 1st quintile widened in 2010 and 2014, while the differences between the 2nd and 3rd quintiles and the 1st quintile reduced from 2008 to 2014 (Table 2). These absolute differences in hospital beds were less than those in health professionals.

Like health resource, the hospitalization rate also significantly increased by 2.269, 3.756, and 4.782 percentage points in 2010, 2012, and 2014 when compared to 2008 in the 1st quintile, as evidenced by Model 3. Statistically, the richer quintiles used significantly more hospital care services. The richer quintiles (2nd, 3rd, 4th, and 5th) were 0.849, 1.202, 1.015, and 0.767 percentage points higher than the poorest quintile. The absolute difference between the 2nd and 3rd quintiles and the 1st quintile decreased following the health care reform (from 2008 to 2014), while the gaps between the 4th and 5th quintiles and the 1st changed little in 2012 and 2014 (Table 2).

## 4. Discussion

Both health resources and access to health care increased in each GDP per capita quintile in rural China from 2008 to 2014. For the distribution, the absolute inequalities in health professionals and hospital beds per 1000 population enlarged, while the absolute inequality in the hospitalization rate kept constant between the richest and the poorest quintile following the new round of health care reform. 

Health professionals are not willing to work in poor areas, even though the government implemented a series of policies in the reform for recruiting and retaining health professionals in poor areas. Personnel expenditure for health workers in the western less-developing areas was 66,000 Chinese yuan (9566 US dollars) in 2014, which was 28% lower than health workers in the eastern well-developing areas (92,000 Chinese yuan, 13,335 US dollars) [13]. In addition to a higher salary, many other factors may affect their willingness to work in poor counties, such as working conditions and career development opportunities [22]. The poor rural counties face more financial difficulties than the rich ones, thus it is harder to retain highly qualified health professionals in the poor areas. It seems that more time is needed for a significant change in the future [6,7]. In terms of the hospital beds, they are closely associated with health professionals. Chinese central government made a guiding policy that the local governments should allocate six hospital beds per 1000 population, which is expected to be realized in 2020 [1]. Besides, the central and local governments invested more fiscal payment to the western and poor areas. Health expenditure (except for personnel expenditure) in the western poorer areas increased 2.3 times in 2014 compared to 2008, and health expenditure increased less than twice in the eastern richer areas [12,13]. Thus, increasing the quantity of hospitals beds is easier than increasing the number of health professionals, but the gaps between the richer and the poorest are still enlarging significantly.

Health access may be related to health resources [23], which contains health financing, health workforce, health infrastructures, etc. China achieved universal health insurance coverage in the rural area in 2010, which had a positive impact on health care utilization, especially in poor rural counties [24]. The new rural cooperative medical scheme (NCMS) was initiated in 2003 and received lots of subsidies by both the central and the local governments. NCMS increased financial access to health care, especially access to inpatient care. Expanding NCMS has been one of the priorities in the health care reform and the government investments since 2009. The rapid increase in NCMS fund improves its capability in financial protection [25]. NCMS is the major explanation for the improved access to health care in poor areas, as found in this study, which is consistent with many other studies [8,15,25,26]. However, as the inequalities of health professionals and hospital beds still exist, inequalities of health access also need to be improved. In addition, we need to notice from our findings that hospital care utilization is still lower in the poorer counties than in the richer counties, which was not expected. The poorer counties must have more health utilization in rural China if an equitable and accessible health system is achieved. This is because people living in the poorer areas are always in a poorer health status, and they need to use more health services than the richer areas. This means there is still a long way to go in the future for improving the access to health care in poor areas. 

China’s current health care reform focuses on strengthening the capacity of health systems in financing and health care delivery. The quantity of health resources and health utilization increased following the reform, but the absolute inequalities were not improved. For further progress, more efforts are needed, including stronger incentives for the health professionals who work in the remote and poor counties, targeting the precise poor areas to allocate the governments’ health subsidies for improving working conditions, as well as sustaining and extending the benefit scope of the NCMS in poor counties [27]. 

There were several limitations in this study. First, this study did not analyze all possible factors that may contribute to the inequalities of health resources and health care due to data availability. Second, health resources and hospital care utilization were measured based on the permanent residents in each of the rural counties. This may have caused bias in the analysis because of the floating population across the counties and regions. In further studies, we will explore the related factors of the inequalities in health resources and health access based on the health system.

## 5. Conclusions

Absolute inequalities of health resources increased, while absolute inequalities of health utilization remained constant following China’s health care reform. The reform needs to continually recruit qualified health workers and appropriately allocate health infrastructures to strengthen the capacity of the health care system in the impoverished areas.

## Figures and Tables

**Table 1:** The number of counties and covered population by income.

GDP per Capita Quintile	2008		2010		2012		2014	
	Number of Counties	Number of the Population (10,000)	Number of Counties	Number of the Population (10,000)	Number of Counties	Number of the Population (10,000)	Number of Counties	Number of the Population (10,000)
1st quintile (The poorest)	375	15,816	362	14,109	378	18,045	396	17,950
2nd quintile	375	19,026	362	18,173	378	19,868	395	19,572
3rd quintile	376	18,918	361	17,814	377	19,057	396	20,015
4th quintile	375	17,935	362	17,615	378	18,176	395	18,827
5th quintile (The richest)	375	17,676	362	17,707	378	18,018	396	19,375
Total	1876	89,371	1809	85,418	1889	93,164	1978	95,739

**Table 2:** The fixed effect model on the density of health professionals, the density of hospital beds, and the hospitalization rate.

Dependent Variable	Model 1	Model 2	Model 3
	The Density of Health Professionals	The Density of Hospital Beds	The Hospitalization Rate
GDP per capita quintile		
2nd	0.028	0.161 ***	0.849 ***
3rd	0.152 *	0.271 ***	1.202 ***
4th	0.043	0.175 *	1.015 ***
5th	−0.130	0.162	0.767 **
*Year*			
2010	0.308 ***	0.468 ***	2.269 ***
2012	0.498 ***	0.892 ***	3.756 ***
2014	0.865 ***	1.328 ***	4.782 ***
GDP per capita quintile * year		
2nd *year of 2010	0.033	−0.050	−0.755 ***
2nd *year of 2012	0.078	−0.088 *	−0.700 ***
2nd *year of 2014	0.155 **	−0.014	−0.649 ^**^
3rd *year of 2010	−0.003	−0.131 ***	−0.986 ***
3rd *year of 2012	0.165 ***	−0.115 **	−0.631 **
3rd *year of 2014	0.151 **	−0.126 *	−1.073 ***
4th *year of 2010	0.068	0.136 ***	−1.085 ***
4th *year of 2012	0.385 ***	0.092	−0.401
4th *year of 2014	0.532 ***	0.193 *	−0.122
5th *year of 2010	0.249 ***	−0.020	−0.659 ***
5th *year of 2012	0.667 ***	0.204 ***	−0.127
5th *year of 2014	0.754 ***	0.169 **	−0.152
Intercept	2.797 ***	2.163 ***	6.270 ***
sigma_u	1.634	1.397	4.256
sigma_e	0.707	0.695	2.272
rho	0.842	0.802	0.778

* *p* < 0.1; ** *p* < 0.05; *** *p* < 0.01.
